# Bidirectional Introgressive Hybridization between a Cattle and Human Schistosome Species

**DOI:** 10.1371/journal.ppat.1000571

**Published:** 2009-09-04

**Authors:** Tine Huyse, Bonnie L. Webster, Sarah Geldof, J. Russell. Stothard, Oumar T. Diaw, Katja Polman, David Rollinson

**Affiliations:** 1 Institute of Tropical Medicine, Department of Parasitology, Antwerpen, Belgium; 2 Laboratory of Animal Diversity and Systematics, Katholieke Universiteit Leuven, Leuven, Belgium; 3 Wolfson Wellcome Biomedical Laboratories, Department of Zoology, The Natural History Museum, London, United Kingdom; 4 Institut Sénégalais de Recherches Agricoles (ISRA), Bel Air, Dakar, Sénégal; Case Western Reserve University, United States of America

## Abstract

Schistosomiasis is a disease of great medical and veterinary importance in tropical and subtropical regions, caused by parasitic flatworms of the genus *Schistosoma* (subclass Digenea). Following major water development schemes in the 1980s, schistosomiasis has become an important parasitic disease of children living in the Senegal River Basin (SRB). During molecular parasitological surveys, nuclear and mitochondrial markers revealed unexpected natural interactions between a bovine and human *Schistosoma* species: *S. bovis* and *S. haematobium*, respectively. Hybrid schistosomes recovered from the urine and faeces of children and the intermediate snail hosts of both parental species, *Bulinus truncatus* and *B. globosus*, presented a nuclear ITS rRNA sequence identical to *S. haematobium*, while the partial mitochondrial *cox1* sequence was identified as *S. bovis*. Molecular data suggest that the hybrids are not 1st generation and are a result of parental and/or hybrid backcrosses, indicating a stable hybrid zone. Larval stages with the reverse genetic profile were also found and are suggested to be F1 progeny. The data provide indisputable evidence for the occurrence of bidirectional introgressive hybridization between a bovine and a human *Schistosoma* species. Hybrid species have been found infecting *B. truncatus*, a snail species that is now very abundant throughout the SRB. The recent increase in urinary schistosomiasis in the villages along the SRB could therefore be a direct effect of the increased transmission through *B. truncatus*. Hybridization between schistosomes under laboratory conditions has been shown to result in heterosis (higher fecundity, faster maturation time, wider intermediate host spectrum), having important implications on disease prevalence, pathology and treatment. If this new hybrid exhibits the same hybrid vigour, it could develop into an emerging pathogen, necessitating further control strategies in zones where both parental species overlap.

## Author Summary

Schistosome blood flukes cause significant disease in humans and their livestock in tropical and subtropical regions of the world. They have a two host-life cycle with a sexual stage within the mammalian host and are transmitted through water contact. Understanding the biology of these dioecious parasites is essential for developing strategies for control of schistosomiasis. Hybridization between schistosome species can occur, but in most cases host specificity and ecology are thought to maintain species barriers. Here, we report on the emergence of a new hybrid strain of schistosome found in northern Senegalese children, resulting from introgressive hybridization between a bovine and human parasite. This situation may have arisen due to the increased number of water contact sites commonly used by both cattle and people linked to recent major water development projects. Our findings have come to light due to optimized sampling and genotyping techniques of individual schistosome larval stages. Gene exchange following hybridization can lead to phenotypic innovations that can ultimately lead to the emergence of new diseases. The impact on disease epidemiology is only now unfolding, and it is essential to monitor the situation closely and move swiftly to control this rapidly evolving situation.

## Introduction

The increasing use of molecular techniques in ecological studies has revealed many cases of hybridization and introgression in plants and animals [1]–[3], but examples in metazoan parasites are rare [2]. Hybridization can have a major impact on adaptive radiation and diversification of the species under study [2],[4], and in the case of parasites, this may also have an impact on the host and the epidemiology of disease. The acquisition of new genes may generate new phenotypes that might differ in virulence, resistance, pathology, and host use, ultimately leading to the emergence of new diseases.

Schistosomiasis is a disease of medical and veterinary importance in tropical and subtropical regions, caused by parasitic flatworms of the genus *Schistosoma* (subclass Digenea). Over 200 million people are infected, of which 85% live in Africa. Chronic infection may lead to severe liver, intestinal and bladder complications, sometimes leading to death. *Schistosoma* species have a two-host life cycle with an asexual stage within an intermediate freshwater snail host and a sexual stage within the definitive mammalian host; parasite eggs are voided in the urine or faeces depending on the species. Schistosomes are highly unusual trematodes as they are dioecious, which creates an opportunity for interplay between male and female parasites in the vasculature system of the definitive mammalian host. Several crossing experiments have been carried out in controlled laboratory settings especially between species belonging to the *Schistosoma haematobium* group (e.g. [5]–[7]). Depending on the phylogenetic distance between the two species involved, crossing may lead to parthenogenesis or hybridization, with certain combinations being more viable than others. Experimental hybrids tend to show heterosis: they have a higher fecundity, faster maturation time, higher infectivity, and wider intermediate host spectrum compared to their parental species (e.g. [7],[8]).

In nature, the distribution of schistosome species and their intermediate snail hosts, together with definitive host specificity are believed to restrict these hybridization events from occurring. However, both natural (climate change) and anthropogenic changes (migration, deforestation, water development) can break down the ecological isolation barriers by facilitating the introduction of parasite species and strains into new areas, resulting in novel host-parasite and parasite – parasite interactions. For example, deforestation of the tropical rainforest in Loum (Cameroun) led to the establishment of the snail *B. truncatus*, the intermediate host of *S. haematobium*. Shortly after, the parasite *S. haematobium* became established. The epidemiology of schistosomiasis changed dramatically: prevalences and infection intensities increased and a complete switch from intestinal schistosomiasis (*S. guineenis*) transmitted by *B. forskalii* to urinary schistosomiasis (*S. haematobium* and the hybrid *S. haematobium*×*guineenis*) was observed [9],[10]. The extent of hybridization only became clear after the application of molecular genetic markers including SSCP and sequence analysis [11]. Other examples of hybridization events that came to light through molecular studies include the hybridization of a primarily human parasite, *S. mansoni* and a rodent parasite *S. rodhaini* in western Kenya [12],[13]. In this example, hybrid cercariae have been recorded from naturally infected *Biomphalaria* snails, but transmission of these hybrids into humans has not been detected.

Because of their inaccessibility in the human mesenteric system, it is very difficult to obtain adult schistosome worms. Traditionally, schistosome infections were identified by egg morphology, site of infection and snail compatibility studies but these methods are not sufficiently sensitive and interactions between species can be missed. Laboratory passage of natural miracidia into adult worms enabled the development of molecular techniques but this labour-intensive technique has a low success rate and leads to artificial selection of specific parasite genotypes [14]. The recent development of a storage protocol for larval schistosomes on Whatman FTA® Cards [15] together with new molecular markers have provided tools to genotype individual miracidia and cercariae sampled directly from the field.

The initial project aim was to perform a large molecular genotyping study of human schistosomes in northern Senegal to study the population structure and transmission dynamics of *Schistosoma* species. The habitats found in the Senegal River Basin (SRB) have changed dramatically over the last 30 years due to the construction of the Diama Dam, intended to prevent salt-water intrusion from the sea facilitating rice and sugar cane agriculture, and the Manantali Dam in Mali, constructed for hydroelectricity and regulation of water flow. The subsequent ecological changes (e.g pH and salinity) together with increased irrigation created new aquatic habitats, which allowed the prolific spread of *Biomphalaria pfeifferi*, the intermediate host of *S. mansoni*, and various species of *Bulinus* responsible for transmitting *S. haematobium* and *S. bovis*. This resulted initially in a major outbreak of human intestinal schistosomiasis (*S. mansoni*) [16],[17] followed by subsequent dynamic changes in the prevelance of both urinary (*S. haematobium*) and intestinal schistosomiasis (Polman, unpublished data). Several areas of sympatry between these schistosomes now exist, and many children can be found with both urinary and intestinal schistosomiasis. It is likely that the prevalence of the bovine schistosomes, especially *S. bovis*, has also increased, but this has not been documented.

Both the laboratories of the Natural History Museum and the Institute of Tropical Medicine involved in the studies discovered independently the occurrence of schistosome miracidia from children with a *S. bovis* mitochondrial genetic profile. In 1970, Taylor demonstrated the successful experimental hybridization of *S. haematobium* and *S. bovis* while Brémond et al. [18] found schistosome species in Niger with allozyme profiles that were intermediate between *S. bovis* and *S. haematobium* or *S. curassoni*, but more sensitive markers were needed to discriminate *S. haematobium* from *S. curassoni*. In this study we use molecular sequencing tools to provide conclusive evidence of the hybridization of *S. haematobium* and *S. bovis* in nature, with hybrid offspring infecting children in northern Senegal. This study provides a first glimpse at the intricacies between a human and a cattle schistosome species and our data are discussed in relation to implications for schistosomiasis epidemiology and control.

## Results

### Nuclear and mt DNA genotyping

#### Sequence identification

*cox1* mtDNA (545 bp) – Pairwise distance analysis of all the sequence data (pure species, hybrids and the reference taxa) showed that there was 12% sequence divergence separating *S. haematobium* and *S. bovis*. The genetic distance between *S. bovis* and *S. curassoni* and between *S. bovis* and *S. mansoni* was 6% and 21% respectively (Table 1). No sequence variation was found within the *S. haematobium* sequences. 0.3% sequence divergence was found within the *S. bovis* sequences. Sequences of the most prevalent hybrids found in the urine and stool samples contained 3 point mutations compared to the sequences from the pure *S. bovis* cercariae. None of the hybrid sequences were identified as being *S. mansoni* or *S. curassoni* (Table 1).

**Table 1:** Uncorrected pair-wise distances between the *cox1* mtDNA sequences of the reference and hybrid schistosomes.

	1	2	3	4	5	6
1. *S. haematobium*	-	-	-	-	-	-
2. *S. bovis*	0.118	-	-	-	-	-
3. *S. bovis cox1* hybrid*	0.114	0.006	-	-	-	
4. *S. haematobium cox1* hybrid**	0	0.118	0.114	-	-	
5. *S. curassoni*	0.118	0.061	0.063	0.118	-	-
6. *S. mansoni*	0.176	0.206	0.202	0.176	0.214	-

Hybrids with the genetic profile: *cox1 S. bovis+ITS rRNA *S. haematobium*; **cox1 S. haematobium+ITS rRNA S. *haematobium*/*S. bovis*.

ITS nuclear rRNA (927 bp) – *S. haematobium* and *S. bovis* differed at 5 point mutations in all specimens examined. No intra-specific variation was detected within all the ITS sequences, with the exception of hybrids 2 and 3 (Table 2). These showed additive chromatogram profiles at the polymorphic positions between the reference *S. bovis* and *S. haematobium* sequences, with the larger peak representing the *S. haematobium* genotype and the smaller detected peak representing the *S. bovis* genotype (peak difference less than 20%).

**Table 2:** Data for the natural *S. haematobium* x *S. bovis* hybrids in northern Senegal.

Location	Stage	From	No. sequenced‡	No. hybrids	Sequence identification	
					*cox1*	ITS rRNA
Nder	Miracidia	Urine	5	1	*S. bovis*	*S. haematobium*
Nder	Miracidia	Urine	5	2	*S. bovis*	*S. haematobium*
Nder	Miracidia	Urine	5	2	*S. bovis*	*S. haematobium*
Nder	Miracidia	Urine	5	2	*S. bovis*	*S. haematobium*
Nder	Miracidia	Urine	5	1	*S. bovis*	*S. haematobium*
Nder	Miracidia	Stool	5	1	*S. haematobium*	*S. haematobium*+*S.bovis* peaks*
Nder	Cercariae	*B. globosus*	49	1	*S. haematobium*	*S. haematobium*+*S.bovis* peaks*
Mbane	Cercariae	*B. truncatus*	13	4	*S. bovis*	*S. haematobium*
Mbane	Eggs	Stool•	16	3	*S. bovis*	*S. haematobium*
Mbane	Miracidia	Urine	11	2	*S. bovis*	*S. haematobium*
Mbane	Eggs	Stool	5	1	*S. bovis*	*S. haematobium*
Mbane	Eggs	Stool	1	1	*S. bovis*	*S. haematobium*
Gaya	Eggs	Stool•	4	2	*S. bovis*	*S. haematobium*
Thiekenne	Eggs	Stool	15	3	*S. bovis*	*S. haematobium*
Thiekenne	Miracidia	Urine	11	3	*S. bovis*	*S. haematobium*
Thiekenne	Miracidia	Urine	4	1	*S. bovis*	*S. haematobium*
Mbodjenne	Eggs	Stool	5	1	*S. bovis*	*S. haematobium*
Tiguet	Eggs	Stool•	5	1	*S. bovis*	*S. haematobium*

*: At the polymorphic positions between the *S. haematobium* and *S. bovis* sequences two significant chromatogram peaks are seen. The higher one matching *S. haematobium* and the lower one matching *S. bovis* (peak heights differing less than 20%).

**•:** Pooled samples from a number of children. All other samples are from individual children.

**‡:** The number of individual larval stages sequenced per patient or snail infected with hybrids.

#### Eggs and miracidia

The majority of eggs and miracidia collected from the human stool and urine samples were identified as *S. mansoni* and *S. haematobium*. However, 15% of the sequenced eggs or miracidia presented a genotype suggesting a hybrid origin. Most of these had an ITS rRNA fragment that was identical to *S. haematobium* and a partial *cox1* mtDNA fragment which was identified as *S. bovis*. A single miracidium (hybrid 2, Table 2) sampled from one of the human stool samples from Nder had a different composition: the *cox1* fragment was identical to *cox*1 from *S. haematobium* and the ITS rRNA sequence showed double chromatogram peaks as described above.

Hybrid eggs and miracidia were found in the urines and stools of 8 and 7 children respectively, in 6 different villages around Lac de Guiers and along the Lower Valley of the Senegal River Basin (Table 2).

#### Cercariae from snails

Cercariae from naturally infected intermediate snail hosts presented pure *S. haematobium* genotypes from *B. globosus* and pure *S. bovis* genotypes from *B. truncatus*. Both snail species were also found shedding cercariae that displayed the hybrid genetic profiles. One out of three infected *B. truncatus* snails collected in Mbane and one *B. truncatus* from Rhenne were shedding hybrid cercariae presenting an ITS sequence identical to *S. haematobium* and a *cox1* sequence identified as *S. bovis*. In Nder, a single cercaria (hybrid 3, Table 2) isolated from *B. globosus* had a *cox1* sequence identical to *S. haematobium* and an ITS sequence identified as both *S. haematobium* and *S. bovis* with double chromatogram peaks as described above.

### Egg morphology

It was not possible to record egg morphology in the field but some limited data are available for samples from Thiekenne. Atypical and intermediate shaped eggs were found in both a pooled stool sample from 6 individual children and a stool sample from a single child. Apart from the expected lateral spined *S. mansoni* eggs three types of terminal spined eggs were observed: the typical *S. bovis* type (length ∼202 µm), the typical *S. haematobium* type (length ∼144 µm) and (variations of) the slender intermediate form between *S. haematobium* and *S. bovis* (length ∼174 µm), suggested to be hybrid eggs [5].

### Co-infections within the definitive host

All but one of the fifteen patients that were infected with hybrids were also infected with *S. mansoni* (median 18 epg) and *S. haematobium* (median 93,5 eggs/10 ml). Several children were found to be passing *S. haematobium* shaped eggs in their stool samples and this could indicate inter-specific mating between *S. mansoni* males and *S. haematobium* females. However most eggs appeared viable and could reflect hybridization between *S. haematobium* and *S. bovis*.

## Discussion

**Figure 1:**
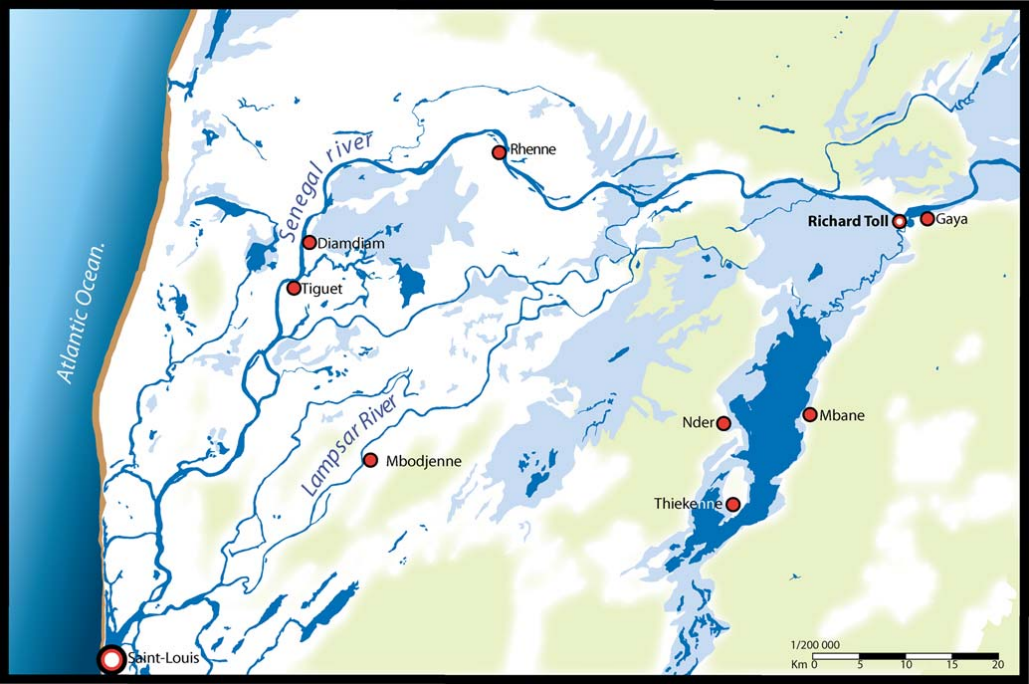
Map of the study area in northern Senegal indicating the villages where *Schistosoma haematobium* x *S. bovis* hybrids have been observed (see Table 2).

Here we present conclusive evidence for the natural hybridization between the cattle parasite *S. bovis* and the human parasite *S. haematobium*. The hybrid species are able to infect humans, and they use the intermediate snail hosts of both parental species, *B. globosus* and *B. truncatus*. Our data imply that one or both of these parental schistosome species has managed to infect the definitive host of its sister species through host switching, enabling these schistosomes to interact. Back-crossing of the hybrid progeny with one of the parental species resulted in the observed introgression. Two hybrid lines have been found, resulting from bidirectional introgressive hybridization. The first line is the most prevalent one resulting from an initial cross between a male *S. haematobium* and a female *S. bovis*, leading to introgression of *S. bovis* mtDNA into *S. haematobium*. This proves that these F1 hybrids are fertile and able to reproduce. The observation of hybrid egg shapes also suggests the occurrence of further generation hybrids and or back-crossing. These data together with the few mutations in the *cox1* fragment compared to the pure *S. bovis* sequence, indicate that this hybridization goes beyond the 1^st^ generation, suggesting a stable ‘hybrid zone’. This is also supported by the occurrence of this hybrid over a wide geographical range, in villages separated from each other by 5–200 kilometer and located near different water resources (different tributaries of the SRB, and Lac de Guiers, see Figure 1). The other hybrid line (resulting from a cross between a male *S. bovis* with a female *S. haematobium*) is much less frequent and is most likely a first generation hybrid as suggested by the double peaks in the nuclear rRNA ITS sequences. F1 hybrids usually display both parental nuclear ITS copies, resulting in additive chromatograms [19],[20]. Biased homogenization towards one of the parental sequences can already occur in F2 hybrids or backcross generations due to concerted evolution operating in the ribosomal arrays [21] or to asymmetrical backcrossing [20]. As such, this cross might be less viable and less successful than the reverse cross. Laboratory experiments have shown that hybridization between schistosomes can be unidirectional, due to competition between males, or due to genomic incompatibility like hybrid breakdown. Taylor [5] showed that experimental crossing between male *S. haematobium* worms and female *S. bovis* worms readily occurred resulting in viable hybrid offspring but he did not test the reverse cross nor backcrosses.

### Hybridization and introgression

**Figure 2:**
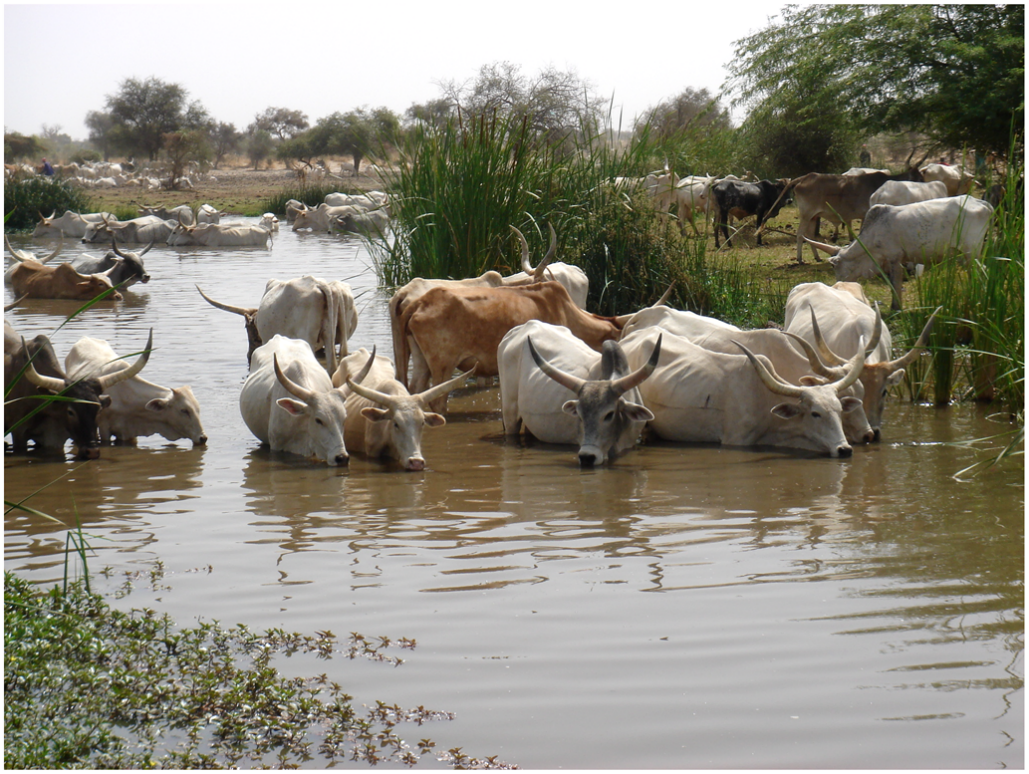
Transmission site in Mbane, Senegal. Hybrid parasites were recovered from snails (*Bulinus truncatus*) collected at this habitat.

Ecological barriers between species can be lost due to both natural and anthropogenic changes. Deforestation of the tropical rainforest in Loum led to the establishment of *S. haematobium* followed by introgressive hybridization and competitive exclusion of the endemic *S. guineensis* [9],[22]. In this particular study, the habitat of the Senegal River Basin has changed dramatically over the last 30 years due to the construction of the Diama and Manantali Dams in Senegal and Mali respectively. These dams prevented salt-water intrusion from the sea and stabilized the flow, facilitating new forms of agriculture. This was followed by human population movements to these resources, increased migration of livestock and snails, creating areas for close associations between humans and domestic livestock facilitating interplay between the schistosomes they carry. In Nder and Thiekenne for example, transmission sites were found where both human and cattle contaminate the water (Figure 2) and where *B. truncatus* and *B. globosus* occur sympatrically. These snail species have very similar diurnal cercarial shedding patterns, potentially facilitating the infection of single definitive hosts with both parasite species [23],[24].

### Host switching and animal reservoirs

The hybridization between *S. bovis* and *S. haematobium* is of special interest as *S. bovis* is a parasite primarily of ruminants and *S. haematobium* a parasite almost exclusively of man. As our findings were discovered during human parasitological surveys we have, as yet, no data from schistosome infections in local cattle, therefore we cannot speculate on whether *S. haematobium* or the hybrid can infect cattle. So, in which host is this initial hybridization occurring? A few studies reported *S. bovis* shaped eggs excreted by man [25]–[27], but it was suggested that this could be due to *S. bovis* eggs passing through the digestive tract of humans eating infected cow livers. Putative *S. haematobium* infections have been documented in primates, rodents and artiodactyls [28],[29]. The identification of these infections has relied heavily on egg morphology alone, thus it is possible that hybrids or other morphological similar species were involved.

The infectivity of a schistosome to a mammalian host depends on the ability of the cercariae to locate the host, penetrate the skin and evade the host's immune system. The phylogeny of the *Schistosoma* genus does not show a single origin of human host use suggesting that host switching may have occurred at several time points in schistosome evolution [30]. Phylogenetically, *S. haematobium* is ancestral to *S. bovis* [30] and the ability to infect humans may have been retained by *S. bovis*. Also, as human skin is thinner than the skin of a bovine, it is perhaps more feasible that *S. bovis* cercariae could penetrate a human rather than *S. haematobium* managing to infect cows. The exact circumstances facilitating hybridization in either cattle or people need to be determined but immunological factors and co-infections with other pathogens will undoubtedly have a role to play.

Another possibility is that the initial pairing between these two schistosome species occurred in a non-human mammalian host that can readily be infected by both species, such as rodents. Rodents are routinely used in laboratories to passage schistosome species, so they are likely to be susceptible in the wild too. However, Duplantier and Sene [31] studied 2000 animals belonging to six different rodent species collected in and around Richard Toll (Senegal) and found only *S. mansoni* in two rodent species (*Arvicanthis niloticus* and *Mastomys huberti*, prevalence of about 5%). Therefore, rodents are unlikely to be involved in this hybridization event, but they might be ideal reservoirs for hybridization between *S. mansoni* and *S. rodhaini* [13]. The role of sheep and goats found in the area does also need to be considered. Neglecting the role of animal reservoirs in transmission might contribute to failure of *Schistosoma* control programmes, as is the case with *S. japonicum* in Asia that infects humans, livestock, rodents and other animals [32],[33].

At the level of the intermediate snail host, the hybrid increased its host range compared to the parental species. Earlier work has shown that *B. truncatus* from the Lower and Middle Valley of the SRB is not susceptible to a *S. haematobium* strain isolated from the Lower Valley, but it is however susceptible to a *S. haematobium* strain isolated from Mali [34], suggesting a strong role for parasite genetics in this host-parasite relationship. The authors also found that natural transmission of *S. haematobium* in the Lower Valley is normally associated with *B. globosus*. In this study we found *B. truncatus* from Mbane and Rhenne (Lower Valley of the SRB) also to be infected with the hybrid species. It appears therefore that hybridization between *S. haematobium* and *S. bovis* is facilitating this breakdown of intermediate snail specificity. This has important implications for the epidemiology of schistosomiasis in northern Senegal because *B. truncatus* became widespread after dam constructions in the early nineties It seems likely that hybridization may well lead to increased transmission intensities in the Senegal River Basin. Given the small sample size of infected snails in this study, additional screening of cercariae is needed to clarify the exact role of each snail species in parasite transmission.

### Distribution

Our data show that the observed hybridization is not a rare isolated phenomenon, hybrids are being found in human populations located near different tributaries of the SRB, and around Lac de Guiers separated by 5–200 km. In 2004, Sene and colleagues [35] described *S. haematobium* from *Bulinus truncatus* collected in Nguidjilone, but our re-inspection of the molecular *cox1* data shows that the genetic profiles are in fact identical to the hybrids found in this present study. Nguidjilone is situated in the Middle Valley of the SRB, more than 600 kilometer further east from our study area.

Suggestions for natural interactions between *S. haematobium* and *S. bovis* were made by Brémond and colleagues [18] who found intermediate egg shapes and intermediate allozyme phenotypes between *S. bovis* and *S. haematobium* or *S. curassoni* collected from children in Niger. Although the allozyme profiles strongly suggested a hybrid origin, the exact role of *S. curassoni* could not be established as the allozyme markers could not discriminate between *S. haematobium* and *S. curassoni*. The authors suggested that *S. curassoni* could also be involved, through initial hybridization with *S. bovis*. Indeed hybridization between *S. bovis* and *S. curassoni* has been reported in Senegal [36] but this study does not find any proof of *S. curassoni* involvement. In either case, this and the above data suggest that hybridization between *S. haematobium* and *S. bovis* is successful in other areas too and that it has been taking place for some time. This ease of hybridization as suggested by the field data and by the experimental work of Taylor [5], together with the fact that hybrid parasites can infect the very abundant *B. truncatus*, suggest this interaction may lead to increased transmission in these areas. This, together with the high intensities that can be reached, indicates the need for further screening and experimental studies (see below).

### Implications

Our findings are of utmost importance due to the possible implications that it bears on the disease dynamics and control strategies for these parasites. The acquisition of new genes through introgressive hybridization can lead to phenotypic innovation that can profoundly influence the evolution of disease. Pitchford and Lewis [37] suggested that the poor response of the cattle parasite *S. mattheei* to oxamniquine treatment might have been due to hybridization with *S. haematobium*, which is not susceptible to the drug. Hybrids between *S. haematobium* and *S. intercalatum* [7],[38] and *S. haematobium* and *S. mattheei* [8] acquired an enhanced infectivity to their laboratory hosts, with increased growth rates and reproductive potential. The natural hybridization of *S. haematobium* and *S. guineensis* in Cameroon had important consequences on the disease dynamics causing severe disease outbreaks in certain areas [9],[10].

If this new hybrid in Senegal exhibits the same hybrid vigour, it can develop into a new emerging pathogen, necessitating new control strategies in zones where both parental species overlap. Given the increased intermediate host range of the hybrid parasite, an intense and rapid control response is required to minimize further spread of the hybrid and possible escalation of human schistosomiasis. The recent increase in urinary schistosomiasis in the villages along the SRB could be a direct effect of hybrid vigour and/or the use of two abundant snail hosts.

### The way forward

To fully understand the consequences of the introgressive hybridization between these two schistosome species further studies are needed. The recent history of *S. haematobium* and *S. mansoni* transmission in northern Senegal is relatively well documented but very little is known about the epidemiology, host use and geographical distribution of *S. bovis*. A study by Diaw et al. [39] reported a sharp increase in bovine schistosomiasis in the lower valley of the SRB, and the appearance of new infection foci from 1989–1990 onwards. It is important to establish the role of cattle, sheep and other possible mammalian reservoir hosts in these hybridization events through genotyping adult worms and larval stages from these possible reservoirs, and also to determine how each snail species is involved in transmission in these foci. This information together with the screening of other localities will provide further insights into the dynamics and the extent of this hybridization.

Experimental infections are needed to study the mating behaviour of *S. bovis* and *S. haematobium* and to study the biological characteristics of the hybrid lines such as fecundity, infectivity, longevity, cercarial production and response to praziquantel, the drug used to treat and control schistosomiasis. If it is not as effective in the hybrid, this can result in more pathology and morbidity.

Genetic introgression is likely to occur in areas of the genome involved in schistosome biology such as virulence, transmission, host specificity and disease processes. When carefully chosen, microsatellite genotyping can provide a genome-wide view of the level of introgression while direct sequencing of targeted gene regions involved in schistosome biology could provide information for potential drug targets and genes involved in isolating mechanisms, speciation and evolution of the *Schistosoma* genus.

## Materials and Methods

### Ethics statement

This study is part of a larger investigation of schistosomiasis epidemiology, transmission and control in Senegal, for which approval was obtained from the ethical committees of the Ministry of Health in Dakar, Senegal, the Institute of Tropical Medicine in Antwerp, Belgium, and the NHS-LREC of Imperial College London, England. According to common practice, all parents and teachers gave oral consent for urine and stool examination and the data were analyzed anonymously. All schistosomiasis positive children were treated with a single dose of praziquantel, at 40 mg/kg of bodyweight. In schools or classes where the percentage of *S. haematobium* or *S. mansoni* infections were more than 50%, mass treatment of all children was carried out at the end of the study.

### Parasite collection

Parasitological surveys were carried out in March 2006 and 2007 in six villages within the Senegal River Basin (SRB) separated by about 10 to 150 miles; Tiguet and Gaya along the Senegal River, Mbodjenne on the Lampsar River and Mbane, Thiekenne, Nder on the shores of the Lac de Guier (see Figure 1, 2). To detect the prevalence of urinary and intestinal schistosomiasis, urine and stool samples were taken from 75 school age children per village, with the exception of Nder where 200 children were involved. For *S. haematobium* detection, 10 mls of each urine sample was filtered using a Nucleopore filter, eggs were viewed and counted under a microscope. Eggs from positive samples were hatched by sedimentation and subsequent exposure to clean water and light. *S. mansoni* infections were diagnosed by duplicate 41.7 mg Kato Katz for each stool sample. Eggs from positive samples were hatched by homogenising each sample with 0.85% saline solution and then passing it through a metal sieve of 212 µm pore size to remove any larger debris. The remaining aqueous solution containing the eggs was then passed through a home-made Pitchford and Visser funnel [40] and washed copiously with bottled mineral water. The eggs were then concentrated within the Pitchford and Visser funnel and placed in a petri dish where they were exposed to clean water and light.

Using a binocular microscope individual miracidia and un-hatched eggs were collected using a Gilson pipette. Where possible egg morphology was recorded by description but no photographs could be taken. *S. mansoni* eggs have a characteristic lateral spine where as *S. bovis* and *S. haematobium* have terminal spines with species specific body shape. Un-hatched eggs were given a further opportunity to hatch by pippetting them into a dish containing fresh water before they were subsequently collected. All samples were individually pipetted onto Whatman FTA® indicator cards in a volume of 3 µl of water and the cards were allowed to dry for 1 hour. Snails caught at the transmission sites were placed into pots containing clean water and exposed to light to stimulate them to shed. Individual cercariae were loaded onto Whatman FTA® indicator cards as described above and the species of snail was identified using morphological characters and recorded. DNA analysis of the FTA samples was carried out at the Natural History Museum in London (Nder material) and the Katholieke Universiteit of Leuven (all other material).

### FTA DNA extraction

A 2.0 mm disc was removed with a Harris Micro Punch from the Whatman FTA® indicator cards at the centre of where the sample was loaded and purified according the manufacturers protocol. Samples were air dried at 56°C for 30 –60 mins. Individual discs were used directly in the multiplex PCR.

### Primary detection of hybrids

This study initially started as routine analysis of *S. haematobium* and *S. mansoni* populations using the partial *cox1* mtDNA marker with the same PCR primers and conditions as described below. Both the NHM and KUL laboratories involved in the studies discovered unusual *cox1* sequences from several eggs and miracidia collected from both urine and stool samples identified as *S. bovis*. The standard Whatman FTA® indicator cards used in our sampling enable the collection and preservation of large numbers of individual larval schistosome stages under field conditions. However, one drawback is that the FTA punch can only be used once, in a single PCR. Therefore, to detect the hybrids, the nuclear ITS rRNA+*cox1* mitochondrial (mt) DNA multiplex PCR was developed for simultaneous amplification of both DNA regions from an individual larval stage (*i.e.* a single FTA disc).

### Multiplex PCR and sequencing at the KUL

The complete ITS rRNA (981 bp)+partial *cox1* mtDNA (585 bp) multiplex PCR amplifications were performed in a total reaction volume of 25 µl and consisted of 1× PCR buffer (Eurogentec), 1.5 mM MgCl_2_ (Eurogentec), 200 µM of each dNTP (Amersham Pharmacia Biotech, Sweden), 1 µM of each primer (Eurogentec; Asmit1 TTTTTTGGTCATCCTGAGGTGTAT [41] and Schisto 3′ TAATGCATMGGAAA-AAAACA [42], ITS4: TCCTCCGCTTATTGATATGC and ITS5: GGAAGTAAAAGTCGTAACAAG [43], 1 unit *Taq* polymerase (Eurogentec), milli-Q water and the individual purified FTA disc. PCR parameters were 3 min at 96°C followed by 40 cycles of 30 sec at 95°C, 30 sec at 54°C and 1 min at 72°C, followed by a final cycle at 72°C for 7 min. The PCR products were visualized on a 1.5% ethidium bromide agarose gel, gel extracted and purified by means of GFX columns according to the manufacturer's instructions (Amersham Pharmacia). The purified products were sequenced using a Big Dye Chemistry Cycle Sequencing Kit (version 1.1) in a 3130 DNA Analyzer (Applied Biosystems), using the original PCR primers.

### Multiplex PCR and sequencing at NHM

The complete ITS rRNA (981 bp)+partial *cox1* mtDNA (585 bp) multiplex PCR amplifications were performed in a total reaction volume of 25 µl using Ready-to-go PCR Beads (Amersham Pharmacia Biotech) each containing 1.5units DNA Taq Polymerase, 10 mM Tris-HCl (pH 9), 50 mM KCl, 1.5 mM MgCl2, 200 µM of each dNTP and stabilizers including BSA, 10 pmol of each primer (*cox1* primers as above) and ITS primers - ITSF: TAACAAGGTTTCCGTAGGTGAA, ITSR: TGCTTAAGTTCAGCGGGT [44] and the individual purified FTA disc. Thermal cycling was performed in a Perkin Elmer 9600 Thermal Cycler and the PCR conditions used were: 5 min denaturing at 95°C: 40 cycles of 30 sec at 95°C, 30 sec at 40°C, 1 min at 72°C; followed by final extension period of 7 min at 72°C. 25 µl of each amplicon were run out on 1.5% Ethidium bromide agarose gel until good separation of the ITS and *cox1* bands could be seen using the UVP system.

Each ITS and *cox1* band was gel extracted and purified using Qiagen PCR gel extraction Kits (Qiagen) according to the manufacturer's protocol. Each band was sequenced with the original PCR primers using a Fluorescent Dye Terminator Sequencing Kit (Applied Biosystems) and the sequencing reactions were run on an Applied Biosystems 377 automated sequencer.

The sequences were assembled and manually edited using Sequencher ver 4.5 (GeneCodes Corp.). Identity of the sequence was confirmed using the Basic Local Alignment Search Tool (BLAST) and by comparison with reference alignments. At the polymorphic positions of the ITS between *S. haematobium* and *S. bovis* any occurrence of double chromatogram peaks was recorded to identify mixed ITS sequences. Sequences are submitted to Genbank under Ac. Nos. FJ588850-62.

### Reference samples/Controls (NHM)

Genomic DNA from schistosomes from Senegal stored in the NHM liquid nitrogen collection was extracted using the DNeasy extraction kit (Qiagen). *S. haematobium* from Guede Chantier, *S. mansoni* from Richard Toll, *S. bovis* from St. Louis and *S. curassoni* from Guede Chantier were used. *S. curassoni* was included to rule out any possible involvement in our findings. The *cox1* and ITS sequences were amplified from the reference samples as described above and these sequences were used as templates to identify and compare the samples in this study. The pair-wise uncorrected distances between the *cox1* sequences were calculated using MEGA 4.1 [45].

## References

[1] Arnold M (1997). Natural Hybridization and Evolution.

[2] Arnold ML (2004). Natural hybridization and the evolution of domesticated, pest and disease organisms.. Mol Ecol.

[3] Mallet J (2005). Hybridization as an invasion of the genome.. Trends Ecol Evol.

[4] Seehausen O (2004). Hybridization and adaptive radiation.. Trends Ecol Evol.

[5] Taylor M (1970). Hybridization experiments on five species of African schistosomes.. J Helminth.

[6] Southgate VR, Rollinson D, Ross GC, Knowles RJ (1982). Mating-behavior in mixed infections of *Schistosoma haematobium* and *Schistosoma intercalatum*.. J Nat Hist.

[7] Webster BL (2003). On the interactions of *Schistosoma haematobium, S. guineensis* and their hybrids in the laboratory and in the field.

[8] Wright CA, Ross GC (1980). Hybrids between *Schistosoma haematobium* and *S. mattheei* and their identification by isoelectric-focusing of enzymes.. Trans R Soc Trop Med Hyg.

[9] Tchuem Tchuenté LA, Southgate VR, Njiokou F, Njine T, Kouemeni LE (1997). The evolution of schistosomiasis at Loum, Cameroon: replacement of *Schistosoma intercalatum* by *S. haematobium* through introgressive hybridization.. Trans R Soc Trop Med Hyg.

[10] Tchuem Tchuenté LA, Morand S, ImbertEstablet D, Delay B, Jourdane J (1996). Competitive exclusion in human schistosomes: The restricted distribution of *Schistosoma intercalatum*.. Parasitology.

[11] Webster BL, Tchuente LAT, Southgate VR (2007). A single-strand conformation polymorphism (SSCP) approach for investigating genetic interactions of *Schistosoma haematobium* and *Schistosoma guineensis* in Loum, Cameroon.. Parisitol Res.

[12] Morgan JAT, DeJong RJ, Lwambo NJS, Mungai BN, Mkoji GM (2003). First report of a natural hybrid between *Schistosoma mansoni* and *S. rodhaini*.. Journal of Parasitology.

[13] Steinauer ML, Hanelt B, Mwangi IN, Maina GM, Agola LE (2008). Introgressive hybridization of human and rodent schistosome parasites in western Kenya.. Mol Ecol.

[14] Shrivastava J, Qian BZ, McVean G, Webster JP (2005). An insight into the genetic variation of *Schistosoma japonicum* in mainland China using DNA microsatellite markers.. Mol Ecol.

[15] Gower CM, Shrivastava J, Lamberton PHL, Rollinson D, Webster BL (2007). Development and application of an ethically and epidemiologically advantageous assay for the multi-locus microsatellite analysis of *Schistosoma mansoni*.. Parasitology.

[16] Picquet M, Ernould JC, Vercruysse J, Southgate VR, Mbaye A (1996). The epidemiology of human schistosomiasis in the Senegal river basin.. Trans R Soc Trop Med Hyg.

[17] Southgate VR (1997). Schistosomiasis in the Senegal River Basin: before and after the construction of the dams at Diama, Senegal and Manantali, Mali and future prospects.. J Helminth.

[18] Bremond P, Sellin B, Sellin E, Nameoua B, Labbo R (1993). Evidence for the introgression of the human parasite *Schistosoma haematobium* by genes from *Schistosoma bovis*, in Niger.. Comptes Rendus De L Academie Des Sciences Serie Iii-Sciences De La Vie-Life Sciences.

[19] Sang T, Crawford DJ, Stuessy TF (1995). Documentation of reticulate evolution in Peonies (Peonia) using internal transcribed spacer sequences of nuclear ribosomal DNA - implications for biogeography and concerted evolution.. P Natl Acad Sci USA.

[20] Aguilar JF, Rossello JA, Feliner GN (1999). Nuclear ribosomal DNA (nrDNA) concerted evolution in natural and artificial hybrids of *Armeria* (Plumbaginaceae).. Mol Ecol.

[21] Dover GA (1986). Molecular drive in multigene families - how biological novelties arise, spread and are assimilated.. Trends in Genetics.

[22] Tchuem Tchuenté LA, Southgate VR, Jourdane J, Webster BL, Vercruysse J (2003). *Schistosoma intercalatum*: an endangered species in Cameroon?. Trends Parasitol.

[23] Jourdane J, Theron A, Rollinson DS, A.J.G. (1987). Larval development: eggs to cercariae.. The biology of schistosomes. From genes to latrines.

[24] Mouahid A, Mone H, Chaib A, Theron A (1991). Cercarial shedding patterns of *S. bovis* and *S. haematobium* from single and mixed infections of *Bulinus truncatus*.. J Helminth.

[25] McMahon JE (1969). *Schistosoma bovis* eggs in human faeces.. Trans R Soc Trop Med Hyg.

[26] Teesdale CH (1976). Spurious human infections with *Schistosoma bovis*.. Trans R Soc Trop Med Hyg.

[27] Chunge R, Katsivo M, Kok P, Wamwea M, Kinoti S (1986). *Schistosoma bovis* in human stools in Kenya.. Trans R Soc Trop Med Hyg.

[28] Rollinson D, Simpson AJG (1987). The biology of schistosomes. From genes to latrines.

[29] Pitchford RJ (1977). A check list of definitive hosts exhibiting evidence of the genus *Schistosoma* Weinland, 1958 acquired naturally in Africa and the Middle East.. J Helminth.

[30] Webster BL, Southgate VR, Littlewood DTJ (2006). A revision of the interrelationships of *Schistosoma* including the recently described *Schistosoma guineensis*.. Int J Parasitol.

[31] Duplantier JM, Sene M (2000). Rodents as reservoir hosts in the transmission of *Schistosoma mansoni* in Richard Toll, Senegal, West Africa.. J Helminth.

[32] McGarvey ST, Zhou XN, Willingham AL, Feng Z, Olveda R (1999). The epidemiology and host-parasite relationships of *Schistosoma japonicum* in definitive hosts.. Parasitol Today.

[33] Riley S, Carabin H, Belisle P, Joseph L, Tallo V (2008). Multi-host transmission dynamics of *Schistosoma japonicum* in Samar Province, the Philippines.. PLoS Med.

[34] Rollinson D, DeClercq D, Sacko M, Traore M, Sene M (1997). Observations on compatibility between *Bulinus truncatus* and *Schistosoma haematobium* in the Senegal River Basin.. Ann Trop Med Parasitol.

[35] Sene M, Southgate VR, Vercruysse J (2004). *Bulinus truncatus*, hôte intermédiaire de *Schistosoma haematobium* dansle bassin du fleuve Sénégal.. Bull Soc Pathol Exot.

[36] Rollinson D, Southgate VR, Vercruysse J, Moore PJ (1990). Observations on natural and experimental interactions between *Schistosoma bovis* and *Schistosoma curassoni* from West-Africa.. Acta Trop.

[37] Pitchford RJ, Lewis M (1978). Oxamniquine in treatment of various schistosome infections in South-Africa.. South African Medical Journal.

[38] Wright CA, Southgate VR, Vanwijk HB, Moore PJ (1974). Hybrids between *Schistosoma haematobium* and *Schistosoma intercalatum* in Cameroon.. Trans R Soc Trop Med Hyg.

[39] Diaw OT, Vassiliades G, Thiongane Y, Seye M, Sarr Y (1998). Extension des trématodoses du bétail après la construction des barrages dans le bassin du fleuve Sénégal.. Revue Elev Méd Vét Trop.

[40] Pitchford RJ, Visser PS (1975). A simple and rapid technique for quantitative estimation of helminth eggs in human and animal excreta with special reference to *Schistosoma* sp.. Transactions R Soc trop Med Hyg.

[41] Bowles J, Blair D, McManus DP (1992). Genetic variants within the genus *Echinococcus* identified by mitochondrial DNA sequencing.. Mol Biol Parasitol.

[42] Lockyer AE, Olson PD, Ostergaard P, Rollinson D, Johnston DA (2003). The phylogeny of the Schistosomatidae based on three genes with emphasis on the interrelationships of *Schistosoma* Weinland, 1858.. Parasitology.

[43] Barber KE, Mkoji GM, Loker ES (2000). PCR-RFLP analysis of the ITS2 region to identify *Schistosoma haematobium* and *S. bovis* from Kenya.. Am Trop Med Hygiene.

[44] Kane RA, Rollinson D (1994). Repetitive Sequences in the Ribosomal DNA Internal Transcribed Spacer of *Schistosoma haematobium*, *Schistosoma intercalatum* and *Schistosoma mattheei*.. Mol Biol Parasitol.

[45] Tamura KD, J. Nei M, Kumar S (2007). MEGA4: Molecular Evolutionary Genetics Analysis (MEGA) software version 4.0.. Mol Biol Evolution.

